# Pneumococcal Serotypes before and after Introduction of Conjugate Vaccines, United States, 1999–2011^1^

**DOI:** 10.3201/eid1907.121830

**Published:** 2013-07

**Authors:** Sandra S. Richter, Kristopher P. Heilmann, Cassie L. Dohrn, Fathollah Riahi, Daniel J. Diekema, Gary V. Doern

**Affiliations:** Cleveland Clinic, Cleveland, Ohio, USA (S.S. Richter);; University of Iowa Carver College of Medicine, Iowa City, Iowa, USA (K.P. Heilmann, C.L. Dohrn, F. Riahi, D.J. Diekema, G.V. Doern)

**Keywords:** Streptococcus pneumoniae, bacteria, streptococci, serotype, vaccine, conjugate vaccines, pathogenesis, United States

## Abstract

Serotyping data for pneumococci causing invasive and noninvasive disease in 2008–2009 and 2010–2011 from >43 US centers were compared with data from preconjugate vaccine (1999–2000) and postconjugate vaccine (2004–2005) periods. Prevalence of 7-valent pneumococcal conjugate vaccine serotypes decreased from 64% of invasive and 50% of noninvasive isolates in 1999–2000 to 3.8% and 4.2%, respectively, in 2010–2011. Increases in serotype 19A stopped after introduction of 13-valent pneumococcal vaccine (PCV13) in 2010. Prevalences of other predominant serotypes included in or related to PCV13 (3, 6C, 7F) also remained similar for 2008–2009 and 2010–2011. The only major serotype that increased from 2008–2009 to 2010–2011 was nonvaccine serotype 35B. These data show that introduction of the 7-valent vaccine has dramatically decreased prevalence of its serotypes and that addition of serotypes in PCV13 could provide coverage of 39% of isolates that continue to cause disease.

Infections caused by *Streptococcus pneumoniae* include meningitis, pneumonia, bacteremia, bronchitis, sinusitis, and otitis media. The World Health Organization estimated that 50%–60% of the 1.6 million deaths caused by pneumococcal infections in 2005 were in children <5 years of age (*1*). In the United States, ≈39,750 cases of invasive pneumococcal disease and 4,000 deaths occur annually (*2*). Since 1977, immunization with a 14-valent (now 23-valent) polysaccharide vaccine has been available in the United States for persons >2 years of age who have an increased risk for serious pneumococcal disease (*3*). Increasing antimicrobial drug resistance in *S. pneumoniae* during the 1990s highlighted the need for a vaccine with effectiveness in young children (*4*,*5*). In 2000, a 7-valent pneumococcal polysaccharide protein conjugate vaccine (PCV7, Prevnar; Wyeth, New York, NY, USA) for serotypes 4, 6B, 9V, 14, 18C, 19F, and 23F became available in the United States for routine use in all children 2–23 months of age and for children 24–59 months of age who are at increased risk for pneumococcal disease (*6*).

A 30% decrease in the incidence of pneumococcal meningitis in the United States from 1998–1999 through 2004–2005 was attributed to direct vaccine effect and herd immunity, but the percentage of cases caused by non-PCV7 serotype strains, particularly 19A, increased (*7*,*8*). In 2007, the World Health Organization recommended use of PCV7 in all countries as part of routine childhood immunization (*1*), and Centers for Disease Control and Prevention (CDC) (Atlanta, GA, USA) guidance for routine use was revised to include healthy children 24–59 months of age who had not yet completed any recommended PCV7 vaccination schedule (*9*). A second pneumococcal conjugate 13-valent vaccine (PCV13) with 6 additional serotypes (1, 3, 5, 6A, 7F, and 19A) was licensed for use in the United States in March 2010 (*10*).

The large number of pneumococcal serotypes and ability of this organism to switch capsules has made prevention of disease through vaccination challenging. The recognition of new serotypes, 6C and 6D, in 2007 and 2009 brought the total number of known pneumococcal serotypes to 93 (*11*,*12*). A large portion of isolates identified as serotype 6A in the past were likely 6C or 6D. Monitoring of pneumococcal serotypes causing disease provides insight into pathogenesis and guidance for vaccine composition.

Although the effect of pneumococcal vaccine on invasive disease has been documented (*7*,*8*,*13*,*14*), the effect on noninvasive disease is unclear. CDC has conducted longitudinal surveillance for invasive pneumococcal disease by using 10 selected sites since the 1990s, but noninvasive disease has been excluded (*2*). Collection of diagnostic specimens that confirm the etiologic agent causing pneumonia, bronchitis, sinusitis, and otitis media can be difficult. Although isolation of *S. pneumoniae* from a normally sterile site is always considered a pathogen, recovery from a respiratory site may represent colonization. Despite these limitations, the role of surveillance that is not limited to isolates causing invasive disease is shown by recent studies reporting high case-fatality rates for noninvasive serotypes (*15*–*17*).

The purpose of this study was to examine changes in pneumococcal serotypes causing invasive and noninvasive disease in all age groups in the United State from 1999–2000 through 2010–2011. The data examined reflect longitudinal surveillance from 4 periods before and after conjugate vaccine use was implemented.

## Materials and Methods

Clinical isolates of *S. pneumoniae* were collected from 45 medical centers throughout the United States during November 1, 2008–April 30, 2009, and from 43 US centers during October 1, 2010–March 31, 2011, as part of a longitudinal surveillance program. For each study period, 50 unique, consecutive pneumococcal isolates considered by the submitting laboratory to have a major clinical role were requested from each center. Isolates were not limited by patient age or specimen source. Identification of isolates was confirmed by using the bile solubility test after receipt at the central reference laboratory.

Susceptibility testing was performed on 1,750 isolates obtained during 2010–2011 and 1,946 isolates obtained during 2008–2009 by using the Clinical Laboratory and Standards Institute broth microdilution method and interpretive criteria (*18*,*19*). The results of susceptibility testing were analyzed by all 3 categories of the Clinical Laboratory and Standards Institute penicillin interpretative criteria (oral, meningitis parenteral, nonmeningitis parenteral) that have been defined since 2008. Clinical laboratories are instructed to report all 3 interpretations for penicillin results on all specimen types, with 1 exception. For cerebrospinal fluid (CSF) isolates, penicillin results are only interpreted according to the meningitis parenteral breakpoints. Therefore, CSF isolates were excluded from the analysis when nonmeningitis parenteral breakpoints were applied.

The capsular serotype of all isolates was determined by using the Quellung reaction with antisera from the Statens Serum Institut (Copenhagen, Denmark). Identity of nontypeable isolates was confirmed by using DNA probes. Serotype distributions during 2008–2009 and 2010–2011 were compared with those during 2 earlier surveillance periods representing the preconjugate vaccine (1999–2000) and the postconjugate vaccine (2004–2005) periods. This comparison required serotyping to be performed on archived penicillin-susceptible and penicillin-intermediate isolates from the 1999–2000 period (*4*). Serotyping was repeated on serogroup 6 isolates from 1999–2000 (penicillin-resistant isolates) and from 2004–2005 to detect 6C and 6D in collections for which serotyping results were published (*20*,*21*). Susceptibility data have been reported for the 1999–2000, 2004–2005, and 2010–2011 surveillance periods (*4*,*21*,*22*). The statistical significance of differences in serotype distribution for time periods, age groups, and specimen sources was assessed by using the Fisher exact test (2-tailed). This research was approved by the human subjects research review board at the University of Iowa.

## Results

Patient age and specimen source distributions for the pneumococcal isolates obtained during the 4 surveillance periods were similar, with 2 exceptions (Table 1). The percentage of isolates from patients ≤5 years of age decreased from 29% in the preconjugate vaccine period to 20%–21% in the postconjugate period (p<0.001). The percentage of isolates from blood cultures decreased from 31% during 1999–2000 to 23%–25% during the postconjugate vaccine period (p<0.01).

**Table 1:** Patient demographics and penicillin susceptibility for *Streptococcus pneumoniae* isolates, United States, 1999–2011*

Characteristic	No. (%) isolates			
	Before PCV7, 1999–2000, n = 1,506	Post-PCV7, 2004–2005, n = 1,647	Post-PCV7, 2008–2009, n = 1,946	Post-PCV13, 2010–2011, n = 1,750
Medical center location				
Northeast	390 (26)	470 (29)	495 (25)	395 (23)
Midwest	394 (26)	403 (24)	395 (20)	433 (25)
Southeast	224 (15)	281 (17)	343 (18)	340 (19)
Southwest	300 (20)	265 (16)	414 (21)	396 (23)
West	198 (13)	228 (14)	299 (15)	186 (11)
Patient location				
Inpatient	846 (56)	995 (60)	1,056 (54)	987 (56)
Outpatient	654 (43)	624 (38)	817 (42)	734 (42)
Unknown	6 (0.4)	28 (2)	73 (4)	29 (2)
Age, y				
0–5†	442 (29)	328 (20)	417 (21)	367 (21)
6–20	86 (6)	137 (8)	160 (8)	173 (10)
21–64	627 (42)	769 (47)	861 (44)	824 (47)
≥65	336 (22)	350 (21)	425 (22)	376 (21)
Unknown	15 (1)	63 (4)	83 (4)	10 (0.6)
Specimen source				
Blood‡	460 (31)	406 (25)	454 (23)	428 (24)
Cerebrospinal fluid	20 (1)	14 (1)	19 (1)	16 (1)
Other sterile body fluid	–§	16 (1)	31 (2)	–§
Middle ear fluid	125 (8)	126 (8)	118 (6)	110 (6)
Lower respiratory tract	670 (44)	870 (53)	953 (49)	840 (48)
Sinus	44 (3)	91 (6)	144 (7)	147 (8)
Other	187 (12)	124 (8)	227 (12)	209 (12)
Penicillin (all isolates)				
Susceptible (MIC ≤0.06 µg/mL)¶	1,005 (67)	1,112 (68)	1,213 (62)	1,066 (61)
Intermediate (MIC 0.12–1 µg/mL)#	178 (12)	295 (18)	403 (21)	352 (20)
Resistant (MIC ≥2 µg/mL)**	323 (21)	240 (15)	330 (17)	332 (19)
Penicillin (noninvasive isolates, oral breakpoint)				
Susceptible (MIC ≤0.06 µg/mL)	652 (64)	793 (65)	828 (57)	747 (57)
Intermediate (MIC 0.12–1 µg/mL)	127 (12)	221 (18)	329 (23)	279 (21)
Resistant (MIC ≥2 µg/mL)	247 (24)	197 (16)	285 (20)	280 (21)
Penicillin (all invasive isolates, meningitis parenteral breakpoint)				
Susceptible (MIC ≤0.06 µg/mL)	353 (74)	319 (73)	385 (76)	319 (72)
Resistant (MIC ≥0.12 µg/mL)	127 (26)	117 (27)	119 (24)	125 (28)
Penicillin (invasive isolates, CSF excluded, nonmeningitis parenteral breakpoint)				
Susceptible (MIC ≤2 µg/mL)	442 (96)	400 (95)	458 (94)	392 (92)
Intermediate (MIC 4 µg/mL)	18 (4)	22 (5)	26 (5)	35 (8)
Resistant (MIC ≥8 µg/mL)	0	0	1 (0.2)	1 (0.2)

*PCV7, pneumococcal conjugate 7-valent vaccine; PCV13, pneumococcal conjugate 13-valent vaccine. †Significant decrease from 29% in the preconjugate vaccine period to 20%–21% in the postconjugate vaccine period (p<0.001). ‡Significant decrease from 31% in 1999–2000 to 23%–25% in the postconjugate vaccine period (p<0.01). §Other sterile body fluids were not recorded as a separate specimen source category during these 2 periods. ¶Penicillin susceptible: parenteral therapy for meningitis or oral therapy. #Penicillin intermediate: oral therapy, resistant if parenteral therapy for meningitis. **Penicillin resistant: parenteral therapy for meningitis or oral therapy.

The serotype distributions of the 2008–2009 and 2010–2011 isolates in comparison with those during 1999–2000 and 2004–2005 are shown in Table 2. The prevalence of PCV7 serotypes decreased from 55% of all isolates in 1999–2000 to 4% in 2010–2011. The percentage of serotype 19A isolates increased from 2% in 1999–2000 to 22% in 2008–2009 (p<0.001), and there was a slight decrease since introduction of PCV13 to 20% (p = 0.09). The percentages of isolates in 3 other predominant serotypes that are included in or related to PCV13 were also similar in 2008–2009 and 2010–2011: serotypes 3 (8.5% and 9.3%), 6C (7.3% and 8.5%), and 7F (5.8% and 4.9%). The only major serotype that increased from 2008–2009 through 2010–2011 was 35B (4.0% in 2008–2009 to 7.0% in 2010–2011; p<0.001).

**Table 2:** Serotype distribution of *Streptococcus pneumoniae* isolates, United States, 1999–2011*

Serotype	No. (%) isolates

All age groups					Children ≤5 y of age			
Before PCV7, 1999–2000	Post-PCV7, 2004–2005	Post-PCV7, 2008–2009	Post-PCV13, 2010–2011		Before PCV7, 1999–2000	Post-PCV7, 2004–2005	Post-PCV7, 2008–2009	Post-PCV13, 2010–2011
PCV7									
4†	69 (4.6)	34 (2.1)	7 (0.4)	6 (0.3)		14 (3.1)	0	0	0
6B†	157 (10.4)	32 (1.9)	8 (0.4)	11 (0.6)		73 (16.3)	7 (2.1)	0	0
9V†	77 (5.1)	21 (1.3)	5 (0.3)	1 (0.1)		19 (4.2)	3 (0.9)	0	0
14†	151 (10.0)	13 (0.8)	7 (0.4)	1 (0.1)		67 (15.0)	0	1 (0.2)	0
18C†	51 (3.4)	10 (0.6)	2 (0.1)	3 (0.2)		23 (5.2)	0	0	0
19F†	181 (12.0)	116 (7.0)	61 (3.1)	47 (2.7)		69 (15.4)	26 (7.9)	15 (3.6)	7 (1.9)
23F†	135 (9.0)	40 (2.4)	5 (0.3)	3 (0.2)		45 (10.1)	8 (2.4)	2 (0.5)	0
Total	821 (54.5)	266 (16.1)	95 (4.9)	72 (4.1)		310 (70.1)	44 (13.4)	18 (4.3)	7 (1.9)
Additional serotypes in PCV13									
1†	17 (1.1)	12 (0.7)	10 (0.5)	2 (0.1)		3 (0.7)	2 (0.6)	1 (0.2)	0
3†	127 (8.4)	184 (11.2)	165 (8.5)	163 (9.3)		14 (3.1)	23 (7.0)	17 (4.1)	15 (4.1)
5†	0	2 (0.1)	1 (0.1)	1 (0.1)		0	0	1 (0.2)	0
6A	87 (5.8)	78 (4.7)	26 (1.3)	7 (0.4)		35 (7.8)	19 (5.8)	5 (1.2)	1 (0.3)
7F†	21 (1.4)	29 (1.8)	113 (5.8)	86 (4.9)		2 (0.5)	2 (0.6)	14 (3.4)	4 (1.1)
19A†	33 (2.2)	239 (14.5)	434 (22.3)	350 (20.0)		12 (2.7)	83 (25.3)	153 (36.7)	125 (34.1)
Total	285 (18.9)	544 (33.0)	749 (38.5)	609 (34.8)		66 (14.9)	129 (39.3)	191 (45.8)	145 (39.5)
PCV related									
6C	12 (0.8)	37 (2.3)	141 (7.3)	148 (8.5)		2 (0.5)	2 (0.6)	29 (7.0)	32 (8.7)
9N	19 (1.3)	14 (0.9)	31 (1.6)	34 (1.9)		4 (0.9)	1 (0.3)	2 (0.5)	2 (0.5)
23A	10 (0.7)	47 (2.8)	86 (4.4)	81 (4.6)		0	9 (2.7)	18 (4.3)	10 (2.7)
23B	2 (0.1)	25 (1.5)	58 (3.0)	44 (2.5)		0	4 (1.2)	14 (3.3)	11 (3.0)
Other	18 (1.2)‡	36 (2.2)§	22 (1.1)¶	31 (1.8)#		5 (1.1)‡	4 (1.2)§	5 (1.2)¶	1 (0.3)#
Total	61 (4.1)	159 (9.7)	338 (17.4)	338 (19.3)		11 (2.5)	20 (6.1)	68 (16.3)	56 (15.3)
Non-PCV									
11A†	52 (3.5)	101 (6.1)	77 (4.0)	82 (4.7)		3 (0.7)	16 (4.9)	12 (2.9)	14 (3.8)
12F	17 (1.1)	29 (1.8)	8 (0.4)	14 (0.8)		1 (0.2)	2 (0.6)	1 (0.2)	0
15A	7 (0.5)	45 (2.7)	78 (4.0)	53 (3.0)		3 (0.7)	10 (3.0)	16 (3.8)	3 (0.8)
15B†	7 (0.5)	38 (2.3)	39 (2.0)	58 (3.3)		4 (0.9)	12 (3.7)	14 (3.4)	22 (6.0)
15C	12 (0.8)	30 (1.8)	29 (1.5)	43 (2.5)		3 (0.7)	9 (2.7)	13 (3.1)	17 (4.6)
16F	17 (1.1)	38 (2.3)	57 (2.9)	39 (2.2)		0	5 (1.5)	5 (1.2)	5 (1.4)
22F†	36 (2.4)	77 (4.7)	95 (4.9)	84 (4.8)		3 (0.7)	14 (4.3)	10 (2.4)	20 (5.4)
35B	30 (2.0)	73 (4.4)	78 (4.0)	123 (7.0)		6 (1.3)	24 (7.3)	20 (4.8)	39 (10.6)
Other	113 (7.5)**	217 (13.2)††	266 (13.7)‡‡	190 (10.9)§§		19 (4.3)**	41 (12.5)††	45 (10.8)‡‡	33 (9.0)§§
NT	48 (3.2)	30 (1.8)	37 (1.9)	45 (2.6)		13 (2.9)	2 (0.6)	4 (1.0)	6 (1.6)
Total	339 (22.5)	678 (41.2)	764 (39.3)	731 (41.8)		55 (12.4)	135 (41.1)	140 (33.6)	159 (43.3)
All	1,506	1,647	1,946	1,750		442	328	417	367

*PCV7, pneumococcal conjugate 7-valent vaccine; PCV13, pneumococcal conjugate 13-valent vaccine; NT, nontypeable; ch, children. †Serotypes in 23-valent polysaccharide pneumococcal vaccine. ‡Other low prevalence serotypes (no. isolates): in 1999–2000, serotype 7C (2), 9A (8, 2 ch), 9L (2), 18F (2), 18B (4, 3 ch). §In 2004–2005, serotype 6D (1), 7A (1), 7C (8), 9A (5, 1 ch), 9L (5), 18F (4), 18A (3, 1 ch), 18B (5, 1 ch), 19B (3, 1 ch), 19C (1). ¶In 2008–2009, serotype 7C (12, 5 ch), 9A (3), 9L (5), 19B (1), 19C (1). #In 2010–2011, serotype 6D (1), 7B (1 ch), 7C (21), 9A (2), 9L (5), 19B (1). **In 1999–2000, serotype 8 (7), 10A (14, 3 ch), 11B (1), 13 (8, 1 ch), 16A (1, 1 ch), 17F (2, 1 ch), 20 (3), 21 (4, 3 ch), 25A (9, 3 ch), 25F (1), 28F (1), 28A (4), 29 (3, 1 ch), 31 (13), 33F (5, 2 ch), 33A (7, 1 ch), 34 (4), 35F (18, 2 ch), 35C (1), 36 (1), 37 (1), 38 (2), 45 (2, 1 ch), 47 (1). ††In 2004–05, serotype 2 (1), 8 (7), 10F (1), 10A (23, 1 ch), 11D (1), 12B (2), 13 (5), 15F (2), 16A (2, 1 ch), 17F (11), 20 (7, 2 ch), 21 (6, 1 ch), 24F (1), 25A (3, 1 ch), 28F (2), 28A (2), 29 (8, 6 ch), 31 (36, 6 ch), 33F (16, 4 ch), 33A (6, 3 ch), 34 (11, 1 ch), 35F (34, 6 ch), 35A (15, 4 ch), 38 (12, 3 ch), 39 (1 ch), 40 (1), 48 (1 ch). ‡‡In 2008–2009, serotype 8 (13, 1 ch), 10F (1), 10A (28, 4 ch), 10B (1), 11D (1), 13 (3), 16A (1), 17F (28, 6 ch), 20 (14, 1 ch), 21 (20, 9 ch), 22A (1 ch), 25A (6, 1 ch), 28A (2), 29 (7, 2 ch), 31 (35, 2 ch), 32F (1), 33F (23, 2 ch), 33A (17, 3 ch), 33B (3, 1 ch), 34 (23, 4 ch), 35F (25, 6 ch), 35A (2), 35C (2, 1 ch), 37 (3), 38 (3), 42 (3, 1 ch). §§In 2010–2011, serotype 8 (10), 10A (26, 5 ch), 13 (2), 17F (15, 3 ch), 20 (6), 21 (15, 9 ch), 24A (2, 1 ch), 28A (1), 29 (3), 31 (28, 2 ch), 33F (16, 3 ch), 33A (18, 3 ch), 34 (15, 2 ch), 35F (17, 4 ch), 38 (15), 45 (1 ch).

In 1999–2000, PCV7 serotypes were expressed by more isolates from children ≤5 years of age (70.1%) than from other patients (48.0%; p<0.0001). By 2010–2011, only 1 PCV7 serotype (19F) was expressed by 7 isolates (1.9%) from children ≤5 years of age, and all PCV7 serotypes were detected among 65 isolates (4.7%) from the other age groups. The percentage of serotype 19A isolates among patients ≤5 years of age peaked in 2008–2009 at 36.7%, and the percentage was 34.1% in 2010–2011. The prevalence of serotype 19A (34.1% vs. 16.3%; p<0.0001) and 35B (10.6% vs. 6.1%; p = 0.004) isolates was higher and the percentage of serotype 3 isolates (4.1% vs. 10.7%; p<0.0001) was lower for children ≤5 years of age than for other age groups in 2010–2011.

Among 444 isolates from invasive specimens (blood and CSF) in 2010–2011 (Table 3), the predominant serotypes were 19A (18.2%), 7F (12.4%), 3 (9.5%), 6C (7.9%), 22F (6.3%), and 23A (5.2%). Serotype 7F isolates were more commonly recovered from invasive than noninvasive specimens (12.4% vs. 2.4%; p<0.001); serotype 35B isolates were recovered more often from noninvasive than invasive specimens (8.0% vs. 4.3%; p = 0.007). Rates of recovery from invasive and noninvasive specimens were similar for other serotypes. The distribution of major serotypes in isolates recovered during 2010–2011 from lower respiratory tract specimens (19A [18.3%], 3 [9.5%], 6C [9.4%], and 35B [8.0%]) was similar to all specimen sources combined. Serotype 19A isolates were more prevalent among middle ear fluid specimens than among other specimen types (36.4% vs. 18.9%; p<0.001).

**Table 3:** Serotype distribution of *Streptococcus pneumoniae* noninvasive and invasive isolates, United States, 1999–2011*

Serotype	No. (%) isolates								
	Noninvasive					Invasive			
	Before PCV7, 1999–2000	Post-PCV7, 2004–2005	Post-PCV7, 2008–2009	Post-PCV13, 2010–2011		Before PCV7, 1999–2000	Post-PCV7, 2004–2005	Post-PCV7, 2008–2009	Post-PCV13, 2010–2011
PCV7									
4†	20 (1.9)	11 (0.9)	5 (0.4)	2 (0.2)		49 (10.2)	23 (5.3)	2 (0.4)	4 (0.9)
6B†	104 (10.1)	25 (2.1)	5 (0.4)	7 (0.5)		53 (11.0)	7 (1.6)	3 (0.6)	4 (0.9)
9V†	47 (4.6)	12 (1.0)	5 (0.4)	0		30 (6.3)	9 (2.1)	0	1 (0.2)
14†	69 (6.7)	9 (0.7)	5 (0.4)	0		82 (17.1)	4 (0.9)	2 (0.4)	1 (0.2)
18C†	25 (2.4)	6 (0.5)	0	2 (0.2)		26 (5.4)	4 (0.9)	2 (0.4)	1 (0.2)
19F†	144 (14.0)	95 (7.8)	55 (3.8)	41 (3.1)		37 (7.7)	21 (4.8)	6 (1.2)	6 (1.4)
23F†	105 (10.2)	33 (2.7)	4 (0.3)	3 (0.2)		30 (6.3)	7 (1.6)	1 (0.2)	0
Total	514 (50.1)	191 (15.8)	79 (5.5)	55 (4.2)		307 (64.0)	75 (17.2)	16 (3.2)	17 (3.8)
Additional serotypes in PCV13									
1†	9 (0.9)	6 (0.5)	4 (0.3)	0		8 (1.7)	6 (1.4)	6 (1.2)	2 (0.5)
3†	108 (10.5)	145 (12.0)	126 (8.7)	121 (9.3)		19 (4.0)	39 (8.9)	39 (7.7)	42 (9.5)
5†	0	2 (0.2)	0	1 (0.1)		0	0	1 (0.2)	0
6A	56 (5.5)	53 (4.4)	21 (1.5)	4 (0.3)		31 (6.5)	25 (5.7)	5 (1.0)	3 (0.7)
7F†	10 (1.0)	11 (0.9)	38 (2.6)	31 (2.4)		11 (2.3)	18 (4.1)	75 (14.9)	55 (12.4)
19A†	24 (2.3)	163 (13.5)	330 (22.9)	269 (20.6)		9 (1.9)	76 (17.4)	104 (20.6)	81 (18.2)
Total	207 (20.2)	380 (31.4)	519 (36.0)	426 (32.6)		78 (16.3)	164 (37.6)	230 (45.6)	183 (41.2)
PCV related									
6C	10 (1.0)	30 (2.5)	104 (7.2)	113 (8.7)		2 (0.4)	7 (1.6)	37 (7.3)	35 (7.9)
9N	14 (1.4)	12 (1.0)	22 (1.5)	20 (1.5)		5 (1.3)	2 (0.5)	9 (1.8)	14 (3.2)
23A	10 (1.0)	37 (3.1)	65 (4.5)	58 (4.4)		0	10 (2.3)	21 (4.2)	23 (5.2)
23B	2 (0.2)	22 (1.8)	50 (3.5)	36 (2.8)		0	3 (0.7)	8 (1.6)	8 (1.8)
Other	12 (1.2)‡	26 (2.1)§	13 (0.9)¶	23 (1.8)#		6 (2.3)‡	10 (2.3)§	9 (1.8)¶	8 (1.8)#
Total	48 (4.7)	127 (10.5)	254 (17.6)	250 (19.1)		13 (2.7)	32 (7.3)	84 (16.7)	88 (19.8)
Non-PCV									
11A†	39 (3.8)	89 (7.4)	64 (4.4)	71 (5.4)		13 (2.7)	12 (2.8)	13 (2.6)	11 (2.5)
12F	4 (0.4)	4 (0.3)	–	3 (0.2)		13 (2.7)	25 (5.7)	8 (1.6)	11 (2.5)
15A	6 (0.6)	35 (2.9)	67 (4.7)	36 (2.8)		1 (0.2)	10 (2.3)	11 (2.2)	17 (3.8)
15B†	5 (0.5)	27 (2.2)	35 (2.4)	52 (4.0)		2 (0.4)	11 (2.5)	4 (0.8)	6 (1.4)
15C	9 (0.9)	21 (1.7)	24 (1.7)	36 (2.8)		3 (0.6)	9 (2.1)	5 (1.0)	7 (1.6)
16F	11 (1.1)	27 (2.2)	44 (3.1)	31 (2.4)		6 (1.3)	11 (2.5)	13 (2.6)	8 (1.8)
22F†	19 (1.9)	50 (4.1)	53 (3.7)	56 (4.3)		17 (3.5)	27 (6.2)	42 (8.3)	28 (6.3)
35B	28 (2.7)	68 (5.6)	70 (4.9)	104 (8.0)		2 (0.4)	5 (1.2)	8 (1.6)	19 (4.3)
Other	94 (9.2)**	165 (13.6)††	198 (13.7)‡‡	144 (11.0)§§		19 (4.0)**	52 (11.9)††	68 (13.5)‡‡	46 (10.4)§§
NT	42 (4.1)	27 (2.2)	53 (2.4)	42 (3.2)		6 (1.3)	3 (0.7)	2 (0.4)	3 (0.7)
Total	257 (25.0)	513 (42.4)	590 (40.9)	575 (44.0)		82 (17.1)	165 (37.8)	174 (34.5)	156 (35.1)
All	1,026	1,211	1,442	1,306		480	436	504	444

*PCV7, pneumococcal conjugate 7-valent vaccine; PCV13, pneumococcal conjugate 13-valent vaccine; NT, nontypeable; non, noninvasive; inv, invasive. †Serotypes in 23-valent polysaccharide pneumococcal vaccine. ‡Other low prevalence serotypes (no. isolates): in 1999–2000, serotype 7C (2 non), 9A (4 non; 4 inv), 9L (2 non), 18F (1 non; 1 inv), 18B (3 non; 1 inv). §In 2004–2005, serotype 6D (1 non), 7A (1 non), 7C (5 non; 3 inv), 9A (4 non; 1 inv), 9L (4 non; 1 inv), 18F (2 non; 2 inv), 18A (3 non), 18B (5 non), 19B (3 inv), 19C (1 non). ¶In 2008–2009, serotype 7C (9 non; 3 inv), 9A (2 non; 1 inv), 9L (1 non; 4 inv), 19B (1 inv), 19C (1 non). #In 2010–2011, serotype 6D (1 non), 7B (1 non), 7C (15 non; 6 inv), 9A (1 non; 1 inv), 9L (4 non; 1 inv), 19B (1 non). **In 1999–2000, serotype 8 (3 non; 4 inv); 10A (13 non, 1 inv); 11B (1 non); 13 (8 non); 16A (1 non); 17F (2 non); 20 (2 non; 1 inv); 21 (3 non; 1 inv); 25A (7 non; 2 inv); 25F ( 1 non); 28F (1 non); 28A (3 non; 1 inv); 29 (1 non; 2 inv); 31 (12 non; 1 inv); 33F (4 non; 1 inv); 33A (6 non; 1 inv); 34 (4 non); 35F (15 non; 3 inv); 35C (1 inv); 36 (1 non); 37 (1 non); 38 (2 non); 45 (2 non); 47 (1 non). ††In 2004–2005, serotype 2 (1 non), 8 (3 non; 4 inv), 10F (1 inv), 10A (17 non; 6 inv), 11D (1 non), 12B (1 non; 1 inv), 13 (3 non; 2 inv), 15F (1 non; 1 inv), 16A (2 non), 17F (7 non; 4 inv), 20 (4 non; 3 inv), 21 (6 non), 24F (1 non), 25A (2 non; 1 inv), 28F (2 inv), 28A (1 non; 1 inv), 29 (8 non), 31 (32 non; 4 inv), 33F (10 non; 6 inv), 33A (2 non; 4 inv), 34 (9 non; 2 inv), 35F (28 non; 6 inv), 35A (15 non), 38 (8 non; 4 inv), 39 (1 non), 40 (1 non), 48 (1 non). ‡‡In 2008–2009, serotype 8 (6 non; 7 inv), 10F (1 inv), 10A (23 non; 5 inv), 10B (1 inv), 11D (1 non), 13 (2 non; 1 inv), 16A (1 inv), 17F (24 non; 4 inv), 20 (7 non; 7 inv), 21 (19 non; 1 inv), 22A (1 non), 25A (5 non; 1 inv), 28A (2 non), 29 (6 non; 1 inv), 31 (27 non; 8 inv), 32F (1 inv), 33F (11 non; 12 inv), 33A (11 non; 6 inv), 33B (3 non), 34 (18 non; 5 inv), 35F (21 non; 4 inv), 35A (2 non), 35C (2 non), 37 (3 non), 38 (1 non; 2 inv), 42 (3 non). §§In 2010–2011, serotype 8 (4 non; 6 inv), 10A (21 non; 5 inv), 13 (2 non), 17F (13 non; 2 inv), 20 (3 non; 3 inv), 21 (14 non; 1 inv), 24A (2 non), 28A (1 non), 29 (3 non), 31 (24 non; 4 inv), 33F (10 non; 6 inv), 33A (9 non; 9 inv), 34 (10 non; 5 inv), 35F (15 non; 2 inv), 38 (12 non; 3 inv), 45 (1 non).

Penicillin-intermediate and penicillin-resistant *S. pneumoniae* isolates defined by the nonmeningitis parenteral breakpoint (MIC ≥4 µg/mL) were rare for all study periods (4%–8% of invasive non-CSF isolates) (Table 1). All of these invasive isolates in 2010–2011 (n = 36) and 93% (25 of 27) in 2008–2009 were serotype 19A.

The percentage of penicillin-nonsusceptible *S. pneumoniae* (PNSP) (MIC ≥0.12 µg/mL, resistant for meningitis; intermediate and resistant categories for oral therapy of nonmeningitis infections) increased from 33% in 1999–2000 to 39% in 2010–2011 (p<0.001). Predominant PNSP serotypes in 2010–2011 were 19A (41%), 35B (15%), 6C (11%), 23A (8%), 15A (6%), and 19F (4%). The change in distribution of predominant PNSP serotypes over time is shown in the Figure. Serotype 19A isolates increased from 5% of PNSP in 1999–2000 to 29% in 2004–2005 and 46% in 2008–2009, and there was a slight decrease to 41% of PNSP in 2010–2011. (PCV13, which includes this serotype, was introduced in March 2010; see Discussion.) Serotype 35B isolates also increased from 4% of PNSP in 1999–2000 to 15% of PNSP in 2010–2011.

**Figure:**
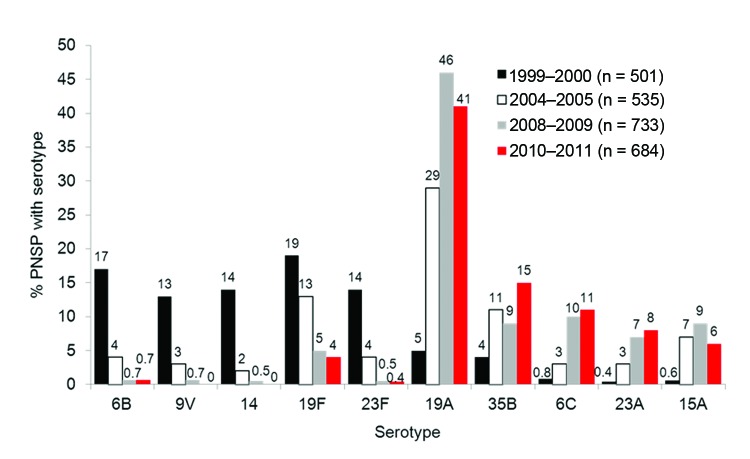
Serotype distribution of penicillin-nonsusceptible *Streptococcus pneumoniae* (PNSP) (MIC >0.12 μg/mL) United States, 1999–2011. Values above bars are percentages.

The rate of penicillin-resistant *S. pneumoniae* (PRSP) (MIC >2 µg/mL, oral therapy) among all isolates decreased from 21% in 1999–2000 (Table 1) to 15% in 2004–2005 (p<0.0001), followed by increases to reach 19% in 2010–2011 (p = 0.0007). Most PRSP isolates during 2010–11 were serotype 19A (75.3%), 35B (11.1%), or 19F (7.2%). Serotype 19A isolates increased from 2% of PRSP in 1999–2000 to 35% in 2004–2005 and 80% in 2008–2009, and there was a slight decrease to 75% of PRSP in 2010–2011. Serotype 35B isolates have also increased from 1% of PRSP in 1999–2000 to 11% of PRSP in 2010–2011.

The rate of penicillin-intermediate *S. pneumoniae* (PISP) (MIC 0.12–1 µg/mL, oral therapy) among all isolates increased from 12% in 1999–2000 to 20% in 2010–2011 (p<0.0001). The most common serotypes of these penicillin-intermediate strains in 2010–2011 were 6C (21%), 35B (19%), 23A (16%), 15A (11%), and 19A (9%).

For each time period, a larger percentage of invasive isolates (72%–76%) were penicillin susceptible (MIC <0.06 µg/mL, meningitis parenteral breakpoint) in comparison to those from noninvasive sites (57%–65%) (Table 1). Separate serotype data for noninvasive and invasive PNSP isolates are shown in Table 4. In 2010–2011, percentages of PCV7 and non-PCV serotypes were higher among noninvasive than invasive PNSP isolates (6.1% vs. 1.6%; p = 0.04 and 32% vs. 21%; p = 0.01), and the prevalence of isolates with additional serotypes in PCV13 was similar (42% and 46%). PCV7-related serotypes comprised a larger percentage of the PNSP invasive isolates (32% vs. 20%; p = 0.004). The prevalence of individual serotypes among 2010–2011 PNSP isolates was similar for noninvasive and invasive isolates, except for 3 serotypes. The percentage of serotypes 23A and 23B among PNSP invasive isolates in 2010–2011 was higher than in noninvasive isolates (15% vs. 6.6%; p = 0.003, and 4.8% vs. 1.3%; p = 0.02). The percentage of serotype 19F among PNSP noninvasive isolates in 2010–2011 was higher than in invasive isolates (4.8% vs. 0.8%; p = 0.04). Serotype distribution over time for noninvasive and invasive isolates obtained from children <5 years of age are shown in Table 5.

**Table 4:** Serotype distribution of pencillin-nonsusceptible noninvasive and invasive pneumococcal isolates, United States, 1999–2011*

Serotype	No. (%) isolates								
	Noninvasive					Invasive			
	Pencillin intermediate and resistant (oral breakpoint), MIC ≥0.12 µg/mL					Pencillin resistant (meningitis breakpoint), MIC ≥0.12 µg/mL			
	Before PCV7, 1999–2000	Post-PCV7, 2004–2005	Post-PCV7, 2008–2009	Post-PCV13, 2010–2011		Before PCV7, 1999–2000	Post-PCV7, 2004–2005	Post-PCV7, 2008–2009	Post- PCV13, 2010–2011
PCV7									
4†	1 (0.3)	2 (0.5)	0	0		1 (0.8)	0	0	0
6B†	63 (16.8)	15 (3.6)	3 (0.5)	4 (0.7)		24 (18.9)	5 (4.3)	2 (1.7)	1 (0.8)
9V†	44 (11.8)	11 (2.6)	5 (0.8)	0		23 (18.1)	7 (6.0)	0	0
14†	38 (10.2)	6 (1.4)	3 (0.5)	0		33 (26.0)	3 (2.6)	1 (0.8)	0
18C†	2 (0.5)	–	0	0		1 (0.8)	0	0	0
19F†	85 (22.7)	56 (13.4)	38 (6.2)	27 (4.8)		10 (7.9)	12 (10.3)	2 (1.7)	1 (0.8)
23F†	58 (15.5)	16 (3.8)	3 (0.5)	3 (0.5)		13 (10.2)	3 (2.6)	1 (0.8)	0
Total	291 (77.8)	106 (25.4)	52 (8.5)	34 (6.1)		105 (82.7)	30 (25.6)	6 (5.0)	2 (1.6)
Additional serotypes in PCV13									
1†	0	1 (0.2)	0	0		0	0	0	0
3†	2 (0.5)	5 (1.2)	0	2 (0.4)		0	1 (0.9)	0	0
6A	17 (4.5)	32 (7.7)	17 (2.3)	3 (0.5)		6 (4.7)	17 (14.5)	2 (1.7)	1 (0.8)
7F†	0	6 (1.4)	0	1 (0.2)		0	0	1 (0.8)	1 (0.8)
19A†	16 (4.3)	111 (26.6)	275 (44.8)	227 (40.6)		7 (5.5)	44 (37.6)	59 (49.6)	55 (44.0)
Total	35 (9.4)	149 (35.6)	292 (47.6)	233 (41.7)		13 (10.2)	62 (53.0)	62 (52.1)	57 (45.6)
PCV-related									
6C	3 (0.8)	12 (2.9)	61 (9.9)	63 (11.3)		1 (0.8)	2 (1.7)	13 (10.9)	14 (11.2)
9A	2 (0.5)	4 (1.0)	2 (0.3)	1 (0.2)		4 (3.2)	0	1 (0.8)	1 (0.8)
19B	0	0	0	1 (0.2)		0	1 (0.9)	1 (0.8)	0
23A	2 (0.5)	13 (3.1)	39 (6.4)	37 (6.6)		0	4 (3.4)	13 (10.9)	19 (15.2)
23B	0	5 (1.2)	12 (2.0)	7 (1.3)		0	0	1 (0.8)	6 (4.8)
Other	2 (0.5)‡	2 (0.5)§	1 (0.2)¶	2 (0.4)#		0	0	0	0
Total	9 (2.4)	36 (8.6)	115 (18.7)	111 (19.9)		5 (3.9)	7 (6.0)	29 (24.4)	40 (32.0)
Non-PCV									
11A†	0	5 (1.2)	2 (0.3)	7 (1.3)		0	1 (0.9)	0	0
15A	3 (0.8)	27 (6.5)	56 (9.1)	29 (5.1)		0	8 (6.8)	9 (7.6)	11 (8.8)
15B†	0	1 (0.2)	6 (1.0)	22 (3.9)		0	0	1 (0.8)	0
15C	0	3 (0.7)	7 (1.1)	12 (2.1)		0	0	3 (2.5)	1 (0.8)
35B	19 (5.1)	56 (13.4)	56 (9.1)	89 (15.9)		1 (0.8)	5 (4.3)	7 (5.9)	13 (10.4)
Other	4 (1.1)**	26(6.2)††	19 (3.1)‡‡	11 (2.0)§§		1 (0.8)**	2 (1.7)††	2 (1.7)‡‡	0
NT	13 (3.5)	9 (2.2)	9 (1.4)	11 (2.0)		2 (1.6)	2 (1.7)	0	1 (0.8)
Total	39 (10.4)	127 (30.4)	155 (25.2)	181 (32.4)		4 (3.1)	18 (15.4)	22 (18.5)	26 (20.8)
All serotypes	374	418	614	559		127	117	119	125

*PCV7, pneumococcal conjugate 7-valent vaccine; PCV13, pneumococcal conjugate 13-valent vaccine; NT, nontypeable; non, noninvasive; inv, invasive. †Serotypes in 23-valent polysaccharide pneumococcal vaccine. ‡Other low prevalence serotypes (no. isolates): in 1999–2000, serotype 9N (1 non), 18 F (1 non). §In 2004–2005, serotype 18B (1 non), 19C (1 non). ¶In 2008–2009, serotype 9N (1 non). #In 2010–2011, serotype 7C (1 non), 9N (1 non). **In 1999–00, serotype 16F (2 non), 22F (1 non), 29 (1 non, 1 inv). ††In 2004–2005, serotype 15F (1 non), 16F (1 non), 20 (1 non), 21 (1 non), 22F (1 inv), 29 (8 non), 31 (1 non), 33F (1 inv), 35A (9 non), 35F (2 non), 39 (1 non), 48 (1 non). ‡‡In 2008–09, serotype 8 (1 non), 11D (1 non), 17F (2 non), 21 (1 non), 22F (1 inv), 22A (1 non), 25A (1 non), 29 (6 non, 1 inv), 33F (1 non), 34 (1 non), 35A (1 non), 42 (3 non). §§In 2010–11, serotype 16F (2 non), 21 (2 non), 29 (1 non), 31 (2 non), 33F (1 non), 34 (1 non), 38 (2 non).

**Table 5:** Serotype distribution of pneumococcal noninvasive and invasive isolates from children ≤5 y of age, United States, 1999–2011*

Serotype	Noninvasive						Invasive				
	Before PCV7, 1999–2000	Post-PCV7, 2004–2005	Post-PCV7, 2008–2009	Post-PCV13, 2010–2011	Pen NS, 2010–2011		Before PCV7, 1999–2000	Post-PCV7, 2004–2005	Post-PCV7, 2008–2009	Post-PCV13, 2010–2011	Pen NS, 2010–2011
PCV7											
4†	4 (1.3)	0	0	0	0		10 (7.0)	0	0	0	0
6B†	46 (15.1)	5 (1.8)	0	0	0		27 (19.0)	2 (3.8)	0	0	0
9V†	14 (4.6)	1 (0.4)	0	0	0		5 (3.5)	2 (3.8)	0	0	0
14†	28 (9.2)	0	1 (0.3)	0	0		39 (27.5)	0	0	0	0
18C†	7 (2.3)	0	0	0	0		16 (11.3)	0	0	0	0
19F†	53 (17.4)	19 (6.9)	14 (3.8)	7 (2.2)	6 (3.2)		16 (11.3)	7 (13.2)	1 (2.0)	0	0
23F†	37 (12.1)	8 (2.9)	2 (0.5)	0	0		8 (5.6)	0	0	0	0
Total	189 (63.0)	33 (12.0)	17 (4.6)	7 (2.2)	6 (3.2)		121 (85.2)	11 (20.8)	1 (2.0)	0	0
Additional serotypes in PCV13											
1†	2 (0.7)	1 (0.4)	0	0	0		1 (0.7)	1 (1.9)	1 (2.0)	0	0
3†	14 (4.6)	22 (8.0)	14 (3.8)	13 (4.0)	0		0	1 (1.9)	3 (6.1)	2 (4.9)	0
5†	0	0	0	0	0		0	0	1 (2.0)	0	0
6A	24 (7.9)	16 (5.8)	5 (1.4)	1 (0.3)	1 (0.5)		11 (7.8)	3 (5.7)	0	0	0
7F†	1 (0.3)	2 (0.7)	6 (1.6)	2 (0.6)	0		1 (0.7)	0	8 (16.3)	2 (4.9)	0
19A†	11 (3.6)	68 (24.7)	135 (36.7)	107 (32.8)	94 (50.3)		1 (0.7)	15 (28.3)	18 (36.7)	18 (43.9)	13 (59.1)
Total	52 (17.3)	109 (39.6)	160 (43.5)	123 (37.7)	95 (50.8)		14 (9.9)	20 (37.7)	31 (63.3)	22 (53.7)	13 (59.1)
PCV-related											
6C	2 (0.7)	2 (0.7)	28 (7.6)	29 (8.9)	21 (11.2)		0	0	1 (2.0)	3 (7.3)	2 (9.1)
9N	4 (1.3)	1 (0.4)	2 (0.5)	2 (0.6)	0		0	0	0	0	0
23A	0	8 (2.9)	17 (4.6)	10 (3.1)	5 (2.7)		0	1 (1.9)	1 (2.0)	0	0
23B	0	4 (1.5)	14 (3.8)	9 (2.8)	1 (0.5)		0	0	0	2 (4.9)	2 (9.1)
Other	4 (1.3)‡	3 (1.1)§	5 (1.4)¶	1 (0.3)#	0		1 (0.7)‡	1 (1.9)§	0	0	0
Total	10 (3.3)	18 (6.5)	66 (17.9)	51 (15.6)	27 (14.4)		1 (0.7)	2 (3.8)	2 (4.1)	5 (12.2)	4 (18.2)
Non-PCV											
11A†	3 (1.0)	15 (5.5)	12 (3.3)	14 (4.3)	4 (2.1)		0	1 (1.9)	0	0	0
15A	3 (1.0)	9 (3.3)	13 (3.5)	2 (0.6)	0		0	1 (1.9)	3 (6.1)	1 (2.4)	1 (4.5)
15B†	3 (1.0)	10 (3.6)	14 (3.8)	20 (6.1)	11 (5.9)		1 (0.7)	2 (3.8)	0	2 (4.9)	0
15C	2 (0.7)	7 (2.6)	11 (3.0)	14 (4.3)	7 (3.7)		1 (0.7)	2 (3.8)	2 (4.1)	3 (7.3)	1 (4.5)
21	3 (1.0)	1 (0.4)	9 (2.5)	9 (2.8)	2 (1.1)		0	0	0	0	0
22F†	2 (0.7)	12 (4.4)	7 (1.9)	18 (5.5)	0		1 (0.7)	2 (3.8)	3 (6.1)	2 (4.9)	0
35B	6 (2.0)	23 (8.4)	20 (5.4)	36 (11.0)	35 (18.7)		0	1 (1.9)	0	3 (7.3)	3 (13.6)
Other	15 (5.0)**	36 (13.1)††	35 (9.5)‡‡	26 (8.0)§§	0		2 (1.4)**	11 (20.8)††	7 (14.3)‡‡	3 (7.3)§§	0
NT	12 (3.9)	2 (0.7)	4 (1.1)	6 (1.8)	0		1 (0.7)	0	0	0	0
Total	49 (16.3)	115 (41.8)	125 (34.0)	145 (44.5)	59 (31.6)		6 (4.2)	20 (37.7)	15 (30.6)	14 (34.1)	5 (22.7)
All	300	275	368	326	187		142	53	49	41	22
% Pen NS	50.2	48.0	59.2	57.4	NA		26.1	43.4	40.8	53.7	NA

*Values are no. (%) isolates unless otherwise indicate. PCV7, pneumococcal conjugate 7-valent vaccine; PCV13, pneumococcal conjugate 13-valent vaccine; Pen NS, penicillin nonsusceptible (MIC ≥0.12 µg/mL); NT, nontypeable; NA, not applicable; non, noninvasive; inv, invasive. †Serotypes in 23-valent polysaccharide pneumococcal vaccine. ‡Other low prevalence serotypes (no. isolates): in 1999–00, serotype 9A (1 non; 1 inv), 18B (3 non). §In 2004–2005, serotype 9A (1 non), 18A (1 non), 18B (1 non), 19B (1 inv). ¶In 2008–2009, serotype 7C (5 non). #In 2010–11, serotype 7B (1 non). **In 1999–2000, serotype 10A (3 non), 12F (1 inv), 13 (1 non), 16A (1 non), 17F (1non), 25A (2 non, 1 inv), 29 (1 non), 33F (2 non), 33A (1 non), 35F (2 non), 45 (1 non). ††In 2004–2005, serotype 10A (1 non), 12F (1 non, 1 inv), 16F (3 non, 2 inv), 16A (1 non), 20 (1 non, 1 inv), 25A (1 inv), 29 (6 non), 31 (5 non, 1 inv), 33F (4 non), 33A (1 non, 2 inv), 34 (1 non), 35F (5 non, 1 inv), 35A (4 non), 38 (1 non, 2 inv), 39 (1 non), 48 (1 non). ‡‡In 2008–2009, serotype 8 (1 non), 10A (4 non), 12F (1 inv), 16F (5 non), 17F (4 non, 2 inv), 20 (1 non), 22A (1 non), 25A (1 non), 29 (1 non, 1 inv), 31 (2 non), 33F (2 non), 33A (2 non, 1 inv), 33B (1 non), 34 (3 non, 1 inv), 35F (5 non, 1 inv), 35C (1 non), 42 (1 non). §§In 2010–2011, serotype 10A (5 non), 16F (5 non), 17F (3 non), 24A (1 non), 31 (2 non), 33F (2 non, 1 inv), 33A (1 non, 2 inv), 34 (2 non), 35F (4 non), 45 (1 non).

## Discussion

This longitudinal study demonstrates the effectiveness of PCV7 in children ≤5 years of age by a decrease in PCV7 serotypes from 70% of isolates in during 1999–2000 to only 1.9% during 2010–2011. Among other age groups, for which routine PCV7 use is not recommended, an indirect vaccine effect is apparent; the percentage of PCV7 serotypes decreased from 48% to 4.9%. Although PCV13 (PCV7 plus serotypes 1, 3, 5, 6A, 7F, and 19A) has been licensed for administration to children and adults >50 years of age, the Advisory Committee on Immunization Practices has not yet issued guidance for use in adults (*23*). Fewer cases and lower cost have been projected as potential benefits if PCV13 were given to the older US population (*24*).

As PCV7 serotypes decreased, serotype 19A strains began causing a higher percentage of invasive and noninvasive disease (*21*,*25*). Serotype 19A was the predominant serotype in our study during the post-PCV periods and accounted for 20% of isolates and 41% of PNSP during 2010–2011. The prevalence of serotype 19A strains was similar for invasive and noninvasive disease. Population-based CDC surveillance of invasive pneumococcal disease in the United States during 2007 reported that 40% of PNSP were serotype 19A (*13*). Recovery of serotype 19A isolates during 2010–2011 from middle ear fluid was much higher in our study than for other specimen sources (36.4% vs. 18.9%). A lower nasopharyngeal carriage rate of serotype 19A in PCV13-vaccinated children in France with acute otitis media suggests a decrease in serotype 19A disease will follow (*26*). Our study showed a slight decrease in the relative number of serotype 19A clinical isolates since introduction of PCV13 that should be apparent as a major trend by the 2012–2013 respiratory season.

The prevalence of serotype 3 strains in this study was fairly constant among invasive and noninvasive specimen types, and there was no evidence of change apparent since PCV13 introduction. High mortality rates have been associated with invasive disease caused by serotype 3 (*27*,*28*). The lower occurrence of serotype 3 isolates among children observed in the present study has also been reported in Germany (*29*).

An increase in 7F strains from 1.4% during 1999–2000 to 5.8% during 2008–2009 (p<0.001) did not change in the post-PCV13 period. A lower rate of nasopharyngeal colonization with 7F strains in children in France vaccinated with PCV13 is predictive of a future decrease in 7F disease (*26*). Serotype 7F was the only serotype recovered in more invasive than noninvasive specimen types in the current study (12.4% vs. 4.9% during 2010–2011). A study in Germany of invasive pneumococcal disease in children <16 years of age demonstrated the highest risk for severe and fatal outcomes for infection with serotype 7F (*27*). However, a meta-analysis associating serotypes with deaths from bacteremic pneumonia, including a small number of cases in children, reported a decreased risk for death for serotype 7F (*28*).

Serotypes 1 and 5 were included in PCV13 because these strains are major causes of invasive disease in children outside the United States (*30*). In our study, only a small number of these serotypes were obtained. During 2010–2011, the 2 serotype 1 isolates were from blood cultures, and the only serotype 5 isolate was from a lower respiratory tract specimen.

Although serotype 6A is included in PCV13, steady decreases in the number of 6A and 6B isolates observed since 1999–2000 suggest cross-reactivity of the 6B PCV7 component against 6A strains. The newly recognized serotype 6C strains increased from 0.8% during 1999–2000 to 8.5% of all isolates during 2010–2011, and a similar change (from 0.5% to 8.7%) was observed in children <5 years of age. Intermediate resistance to penicillin was noted in 50% of the serotype 6C isolates obtained from all age groups and in 72% of 6C strains recovered from the youngest patient age group. Opsonophagocytic killing studies comparing responses of serum from PCV7 and PCV13 recipients to serotype 6C showed minimal response with PCV7, but a strong response was elicited by PCV13 (*31*). Responses to serotype 6A and 6B were strong for immune serum samples from PCV7 and PCV13 recipients (*31*). In the current study, lack of a major increase in the frequency of isolates with serotype 6C during 2010–2011 (8.5%) compared with 2008–2009 (7.3%; p = 0.17) suggests PCV13 cross-reactivity to serotype 6C.

The trend of a relative increase in serotype 35B strains observed in this study is likely to continue because it is not included in PCV13. Serotype 35B isolates were more commonly recovered from children <5 years of age and from noninvasive specimens. Most (83%) of the 35B isolates obtained in 2010–2011 were PNSP. CDC reported that 51% of serotype 35B strains causing invasive disease during 1995–2001 were from older patients (>60 years of age) and 69% were PNSP (*32*).

A limitation of this study is the lack of incidence data. Only relative changes among serotypes causing disease are documented. The proximity in time of 2010–2011 data to PCV13 introduction limited our assessment of the effect of this vaccine.

On the basis of this 2010–2011 surveillance data, PCV13 could provide coverage for 48% of PNSP and 39% of all isolates causing disease. Continued monitoring of pneumococcal serotypes causing invasive and noninvasive disease will be crucial for assessing the full effect of PCV13.
